# Transcriptional activation of the *Lats1 *tumor suppressor gene in tumors of CUX1 transgenic mice

**DOI:** 10.1186/1476-4598-8-60

**Published:** 2009-08-05

**Authors:** Rania Siam, Ryoko Harada, Chantal Cadieux, Robert Battat, Charles Vadnais, Alain Nepveu

**Affiliations:** 1 Goodman Cancer Center, McGill University, 1160 Pine Avenue West, Room 410, Montreal, Quebec H3A 1A3, Canada; 2 Biology Department and The YJ-Science and Technology Research Center, American University in Cairo, Egypt; 3 Department of Biochemistry, McGill University, Montreal, Canada; 4 Department of Oncology, McGill University, Montreal, Canada; 5 Department of Medicine, McGill University, Montreal, Canada

## Abstract

**Background:**

*Lats1 *(large tumor suppressor 1) codes for a serine/threonine kinase that plays a role in the progression through mitosis. Genetic studies demonstrated that the loss of LATS1 in mouse, and of its ortholog *wts *(warts) in Drosophila, is associated with increased cancer incidence. There are conflicting reports, however, as to whether overexpression of *Lats1 *inhibits cell proliferation. CUX1 is a transcription factor that exists in different isoforms as a result of proteolytic processing or alternative transcription initiation. Expression of p110 and p75 CUX1 in transgenic mice increases the susceptibility to cancer in various organs and tissues. In tissue culture, p110 CUX1 was shown to accelerate entry into S phase and stimulate cell proliferation.

**Results:**

Genome-wide location arrays in cell lines of various cell types revealed that *Lats1 *was a transcriptional target of CUX1. Scanning ChIP analysis confirmed that CUX1 binds to the immediate promoter of *Lats1*. Expression of *Lats1 *was reduced in cux1^-/- ^MEFs, whereas it was increased in cells stably or transiently expressing p110 or p75 CUX1. Reporter assays confirmed that the immediate promoter of *Lats1 *was sufficient to confer transcriptional activation by CUX1. *Lats1 *was found to be overexpressed in tumors from the mammary gland, uterus and spleen that arise in p110 or p75 CUX1 transgenic mice. In tissue culture, such elevated LATS1 expression did not hinder cell cycle progression in cells overexpressing p110 CUX1.

**Conclusion:**

While inactivation of *Lats1*/*wts *in mouse and Drosophila can increase cancer incidence, results from the present study demonstrate that *Lats1 *is a transcriptional target of CUX1 that can be overexpressed in tumors of various tissue-types. Interestingly, two other studies documented the overexpression of *LATS1 *in human cervical cancers and basal-like breast cancers. We conclude that, similarly to other genes involved in mitotic checkpoint, cancer can be associated with either loss-of-function or overexpression of *Lats1*.

## Background

The large tumor suppressor (Lats)/Warts (*wts*) gene is conserved from insect to humans and codes for a serine/threonine kinase that was successively implicated as a tumor suppressor, a regulator of mitosis and a key component of the so-called Hippo pathway that controls the coordination between cell proliferation and apoptosis (reviewed in [1,2]). Mutations in the Drosophila lats1 homolog, warts (*wts*), were found originally to cause various developmental defects and dramatic overproliferation phenotypes including the development of tumors in various tissues [3,4]. Complementation assays in flies demonstrated that the human *LATS1 *gene was able to suppress tumour growth and rescue all developmental defects of *wts *mutants including embryonic lethality [5]. *Lats1*-deficient mice spontaneously developed non-metastatic ovarian stromal cell tumours and metastatic large soft tissue sarcomas with a penetrance of 100% and 14%, respectively [6]. Loss-of-heterozygosity (LOH) of the *LATS1 *chromosomal locus at 6q24-25.1 has been reported in a fraction of human breast cancers and salivary duct carcinomas, but inactivating point mutations have not been found [7,8]. Hypermethylation of the *LATS1 *gene promoter and reduction in mRNA expression was documented in some human breast cancers, soft tissue sarcomas and astrocytomas [9-11].

No substrate of LATS1 was known until the hippo pathway was deciphered and a downstream transcriptional effector was identified. The Hippo (Hpo) pathway comprises two kinase/adaptor complexes and a transcription factor whose phosphorylation status controls its sub-cellular localization. In Drosophila, the Hippo/Salvador (Hpo/Sav) complex can phosphorylate and activate WARTS (Wts) [12]. In turn, the (Wts/Mats) complex phosphorylates Yorkie, thereby maintaining it into the cytoplasm [13,14]. Inactivating mutations in hpo, sav, mats and wtz or overexpression of yki produce similar phenotypes such as increased cell proliferation, and diminished apoptosis, as well as tissue overgrowth (reviewed in [1]). Mammals share a similar pathway whereby the Mst2/WW45 phosphorylates LATS1 [15], and the LATS1/Mob1 complex phosphorylates YAP1 to keep it in the cytoplasm [13,16].

LATS1 was localized to centrosomes in interphase cells but associated with the mitotic apparatus during mitosis [17]. The fraction of LATS1 proteins present at the spindle pole bodies during mitosis was shown to be phosphorylated by cyclin B/Cdc2 [18]. LATS1 also associated with zyxin on the mitotic apparatus [19], and colocalized with, and inhibited the activity of, LIMK1 at the actomyosin contractile ring during cytokinesis [20]. These findings provided convincing evidence that LATS1 plays a role in mitosis, however, there are conflicting data as to whether *Lats1 *inhibits or stimulates progression through mitosis. Overexpression of human LATS1 in MEF cells and human tumor cell lines caused the inhibition of Cdc2 kinase activity and this was associated with cell cycle arrest in G2/M or apoptosis [5,21,22]. On the other hand, LATS1 was found to stimulate cytokinesis by negatively regulating LIMK1 and actin polymerization [20]. Moreover, LATS1 belongs to the nuclear Dbf2-related (NDR) family of protein kinases which are involved in cell cycle control in yeast [23]. Indeed, LATS1 forms with MOB1 a complex that localizes at the centrosomes and midbody, and overexpression of LATS1 allowed cells to overcome the spindle checkpoint, whereas siRNA against LATS1 or Mob1A extended telophase, but not other phases of mitosis [24]. These results led the authors to propose that LATS1 is a component of the mammalian mitotic exit network. In this model, the previously described ability of LATS1 to arrest cells in G2 could be explained if the excessively high expression of LATS1 caused the premature inactivation of Cdc2 and, as a consequence, prevented entry into mitosis.

CUX1 (cut homeobox 1) proteins are a family of transcription factors present in all metazoans and involved in the control of many cellular processes including determination of cell identity, cell cycle progression, cell-cell communication and cell motility (reviewed in [25,26]). At least three CUX1 protein isoforms have been described: p200, p110 and p75. The full-length protein, p200 CUX1, makes a rapid but unstable interaction with DNA and is responsible for the CCAAT-displacement activity that has been reported in early studies [27-29]. A shorter isoform, p110 CUX1, is generated as a result of proteolytic processing that does not takes place until the end of the G1 phase in normal cells but becomes constitutive in many transformed cells [30-32]. A third isoform, p75 CUX1, is generated from a shorter mRNA that is initiated within intron 20 and whose expression is restricted to certain cell-types and was found to be activated in some human breast tumors and cancer cell lines [33]. The p75 and p110 isoforms are much less abundant than the full-length isoform, however, in contrast to p200 both CUX1 short isoforms can make a stable interaction with DNA and, depending on promoter-context, can function as transcriptional repressor or activator [30,31,33-35]. Constitutive expression of p110 CUX1 was shown to stimulate cell proliferation by accelerating entry into S phase [36].

Elevated *CUX1 *mRNA and protein expression was reported in primary tumors and cancer cell lines of various types [37-40]. A more detailed analysis revealed that expression of any one of the short isoforms was increased in some tumors and cancer cell lines. The steady-state level of p110 CUX1 was found to be elevated in many human uterine leiomyomas [40]. While proteolytic processing of CUX1 is tightly regulated during the cell cycle in normal cells, it is constitutive in many transformed cells as a result of increased expression of nuclear cathepsin L [30,32]. The intron 20-mRNA and p75 isoform was found to be aberrantly expressed in some primary breast tumors and cancer cell lines [33]. To investigate and compare the oncogenic potential of the 3 CUX1 isoforms, we used specific transgenesis to generate mice carrying a p75, p110 or p200 CUX1 transgene under the control of the mouse mammary tumor virus promoter (MMTV) and integrated into the hypoxanthine phosphoribosyltransferase (hprt) locus. In mixed genetic backgrounds, many virgin p75 CUX1 mice succumbed to myeloproliferative-disease (MPD)-like myeloid leukemias [41]. In the FVB genetic background, multiparous transgenic mice carrying a p75 or p110 CUX1 transgene developed malignancies in the mammary gland and other tissues, with a penetrance of approximately 50% [42]. Overall, these results demonstrated the oncogenic potential of p110 and p75 CUX1.

To identify targets of CUX1 that are involved in cell cycle progression, we recently performed genome-wide location analysis using a promoter microarray and 8 cell lines of various cell-types [35]. Functional classes that were over-represented among targets included genes involved in mitosis and DNA replication initiation [35]. Expression of DNA replication targets was increased in cells over-expressing p110 CUX1, and was reduced following CUX1 knockdown. In contrast to the effect observed on most target genes, the p21^CIP1 ^and cyclin H genes were repressed by p110 CUX1 [35,43]. The promoter-specific repression or activation of target promoters was confirmed in reporter assays [35]. One putative target that was identified in all location arrays is the *Lats1 *gene. In the present study, we performed experiments to verify whether *Lats1 *is a direct target of CUX1 and we investigated the regulatory effect of CUX1 on *Lats1*. We found that p110 and p75 CUX1 can stimulate the transcription of *Lats1 *both in reporter assays and in cell lines stably expressing p110 or p75 CUX1. Moreover, *Lats1 *expression was consistently elevated in tumors of various tissue-types that develop in p110 or p75 CUX1 transgenic mice.

## Results

### *Lats1 *Is Identified As a Putative Target of CUX1 in Cells of Various Tissue Types

**Figure 1:**
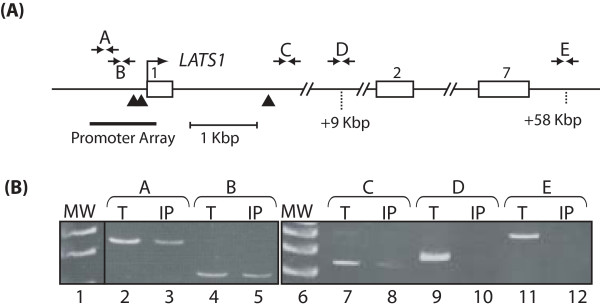
**Scanning ChIP analysis of the *LATS1 *locus using CUX1 antibodies**. (A) Diagram of the *LATS1 *locus. Exons are numbered and the transcription start site is indicated by an arrow. The positions of primer pairs used in the analysis of chipped DNA are indicated by arrows above the map. Primer sequences are given in Materials & Methods. The positions of p110 and p75 CUX1 consensus binding sites (ATCRAT) are indicated by black triangles below the map. The thick line shows the genomic sequences present on the promoter microarray that was used in the location array analysis. (B) Chromatin from Hs578T cells was immunoprecipitated with the CUX1-861 antibody and analyzed by PCR amplification using primers specific for different regions of the *LATS1 *locus. Templates for the PCR reactions were 0.1% total input DNA (T) or ChIP-purified DNA (IP).

Genome location analysis using a promoter array was previously performed in triplicate using chromatin from 8 human cell lines of various cell types: breast tumor (Hs578T), cervical carcinoma (HeLa), kidney epithelial (HEK293), myeloid leukemia (K562, HEL), and B cell lymphoma (Ramos, U266, RPMI8266) ([35] and supplemental data therein). We utilized a genomic DNA microarray containing the region spanning 800 bp upstream and 200 bp downstream of transcription start sites of 18,660 human genes [44]. Strikingly, the *Lats1 *gene was identified as a putative target in all cell lines tested. The p values of the CUX1/*Lats1 *interaction observed in each cell line are shown in Table 1. To confirm that CUX1 binds to the promoter of the *Lats1 *gene, scanning ChIP was performed using primer pairs from different regions of the *Lats1 *locus and chromatin from Hs578T cells immunoprecipitated with CUX1 antibodies. Our results showed that CUX1 bound to the region immediately upstream of the transcription start site and amplified by primer pairs A and B (Fig. 1B, lanes 3 and 5). Note that this region was included within the *Lats1 *promoter sequences present on the promoter microarray (Fig. 1A, see thick line under the map). A weaker interaction was also observed within the first intron (Fig. 1B, lane 8). In contrast, no binding was observed to distal sequences located further downstream of the transcription start site (Fig. 1B, lanes 10 and 12). Interestingly, sequences matching the ATCRAT consensus binding site for the Cut repeat 3 and Cut homeodomain composite DNA binding domain of CUX1 were found at 2 positions upstream of the transcription start site and at one position within intron 1 (Fig. 1A, see black triangles below the map) [45]. Using the primer pair A, binding of CUX1 to the *Lats1 *immediate promoter was validated in all cell lines (data not shown). Altogether, these results demonstrate that CUX1 binds to the immediate *Lats1 *promoter in cells of various tissue types.

**Table 1:** *Lats1 *is identified as a putative target of p110 CUX1 in 8 cell lines

HEL	4.3E-11
K562	3.5E-9

U266	1.5E-3

Ramos	4.0E-4

Hs578T	3.5E-7

HeLa	1.6E-4

293	1.5E-8

RPMI	5.7E-6

Genome-wide location analysis was performed using a panel of 8 human cell lines of various cell types: breast tumor (Hs578T), cervical carcinoma (HeLa), kidney epithelial (HEK293), myeloid leukemia (K562, HEL), and B cell lymphoma (Ramos, U266, RPMI8266). For each cell line, hybridization experiments were done in triplicate using three independent ChAP-enriched and input DNA samples (8 cell lines × 3 hybridizations = 24). ChAP-enriched DNA regions associated with p110 CUX1 were purified and labeled with Cy5 dye. Equal amounts of Cy5-labeled ChAP products and Cy3-labeled input DNA samples were co-hybridized onto a human genomic DNA microarray containing the region spanning 800 bp upstream and 200 bp downstream of transcription start sites of 18,660 human genes. P values observed for the *Lats1 *promoter are shown. The data is taken from [35].

### The immediate promoter region of *LATS1 *is sufficient for the regulation by CUX1

**Figure 2:**
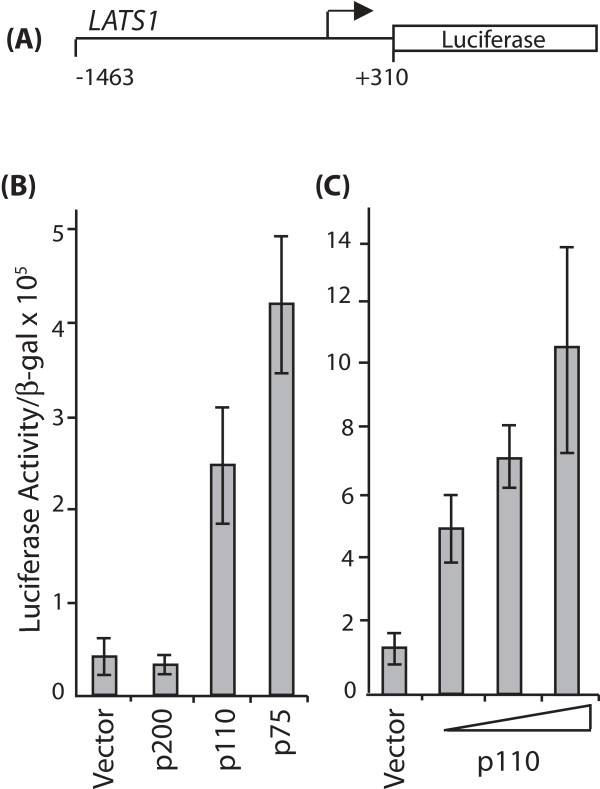
**CDP/Cux p110 and p75 isoforms increases *Lats1 *transcription activity in reporter assays**. (A) Diagram of the *LATS1 *reporter construct. The promoter region of the *LATS1 *gene, from nt -1463 to +310, was cloned into a luciferase reporter plasmid. (B) Hs578T cells were transfected with the *LATS1 *reporter plasmid together with vectors expressing either p200, p110 or p75 CUX1 or with an empty vector. 24 hours after transfection, whole cell extracts were prepared and processed to measure luciferase activity. The results are expressed as relative light units (RLU), normalized to β-galactosidase activity from an internal control and the standard deviation of 3 transfections is shown. (C) NIH3T3 cells were transfected with the *LATS1 *reporter plasmid together with increasing amount of a vector expressing p110 CUX1 or with an empty vector. Samples were processed as detailed in (B).

We wanted to confirm whether the 1-Kbp *LATS1 *genomic region present on the promoter microarray was sufficient for transcriptional regulation by CUX1. The *LATS1 *promoter sequences were introduced into a luciferase reporter plasmid and luciferase activity was measured in the presence of effector plasmids expressing either p200, p110 or p75 CUX1. Previous studies have revealed that the full-length CUX1 isoform, p200, functions as a transcriptional repressor whereas the shorter CUX1 isoform, p110 and p75, can function as repressors or activators depending on promoter-context [30,34,35,43,46]. Expression was not significantly affected by p200 CUX1, but was stimulated in the presence of either p110 or p75 CUX1 (Fig. 2B, left panel). Moreover, the fold-activation by p110 CUX1 was proportional to the amount of effector plasmid (Fig. 2B, right panel). We conclude that the immediate promoter region of *LATS1 *is sufficient for its regulation by p110 and p75 CUX1.

### p110 and p75 CUX1 activate transcription of the endogenous *Lats1 *gene

**Figure 3:**
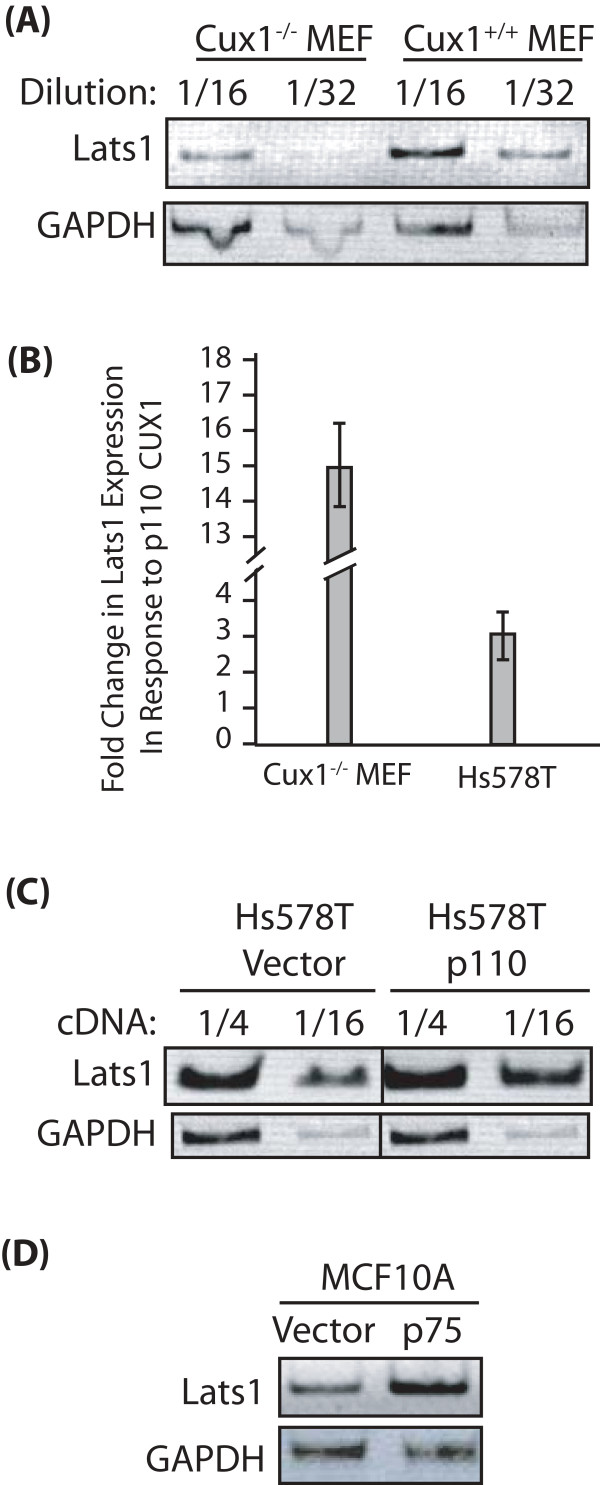
**Endogenous *Lats1 *expression is regulated by CUX1**. RNA was prepared from mouse embryo fibroblasts and various cell lines expressing either p110 or p75 CUX1 or carrying an empty vector, as indicated. *Lats1 *mRNA expression was analyzed by RT-PCR using GAPDH as a control. Amplicons were resolved by agarose gel electrophoresis (A-D). (A) Mouse embryo fibroblasts (MEFs) derived from cux1^-/- ^and cux1^+/+ ^mice. (B) *Lats1 *mRNA expression was measured by quantitative real-time PCR in cux1^-/- ^MEF and Hs578T human breast tumor cells expressing p110 CUX1 or carrying an empty vector. GAPDH levels were used to normalize the samples. The values are the mean of three measurements and error bars represent standard deviation. (C) MCF10A human mammary epithelial cells (D) NMuMG mouse mammary epithelial cells.

We then verified the effect of CUX1 on the endogenous *Lats1 *gene. First, we compared *Lats1 *mRNA expression in mouse embryo fibroblasts (MEFs) derived from a cux1^-/- ^knockout mouse or a wild type littermate [36]. RT-PCR analysis using two dilutions of the mRNA preparations showed that *Lats1 *expression was higher in wild type than in cux1^-/- ^MEFs (Fig. 3A). This result suggested that CUX1 is required for optimal expression of *Lats1*. Next, cux1^-/- ^MEFs were infected with a retroviral vector expressing p110 CUX1 or with a control empty vector and we measured *Lats1 *mRNA expression by quantitative real-time PCR amplification. *Lats1 *mRNA expression was increased on average 15-fold in cux1^-/- ^MEF cells expressing p110 CUX1 (Fig. 3B). We then turned to cell lines that express CUX1 and asked whether increasing the level of p110 or p75 CUX1 would affect endogenous *Lats1 *expression. *Lats1 *mRNA steady-state level was increased approximately 3-fold in Hs578T breast tumor cells that were infected with the p110 CUX1 retroviral vector (Fig. 3B and 3C). Similarly, *Lats1 *expression was increased in the MCF10A human mammary epithelial cell line following infection with a retroviral vector expressing p75 (Fig. 3D). Altogether, these results demonstrate that p110 and p75 CUX1 stimulate expression of the endogenous *Lats1 *gene.

### *Lats1 *is over-expressed in tumors from CUX1 transgenic mice

**Figure 4:**
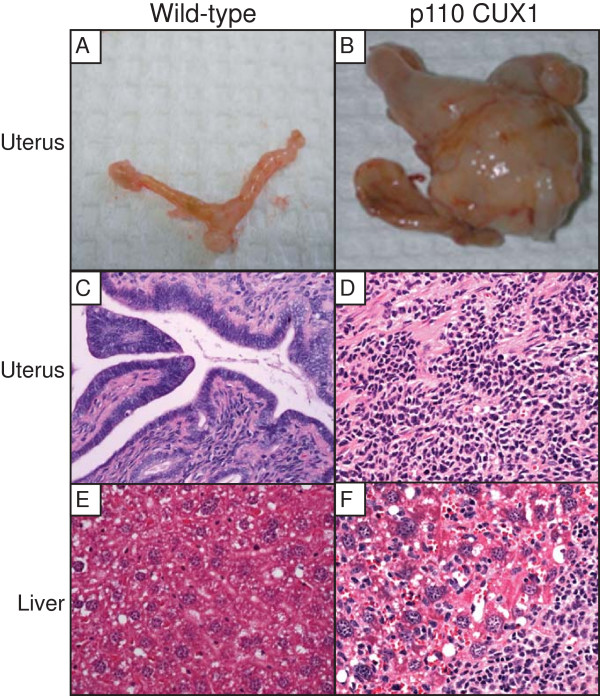
**p110 CUX1 transgenic mice develop uterine histiocytic sarcomas with liver infiltration**. (A, B) Pictures of the uterus from wild-type (A) and p110 CUX1 transgenic (B) mice. (C-F) H&E staining of the uterus (C, D) and liver (E, F) from wild-type and p110 CUX1 transgenic mice respectively.

Transgenic mice have been engineered to express p75 or p110 CUX1 under the control of regulatory sequences from the long terminal repeat of the Mouse Mammary Tumor Virus (MMTV-LTR) [41]. To be able to compare the two CUX1 isoforms, specific transgenesis was used to integrate the transgene into the hypoxanthine phosphoribosyltransferase (hprt) locus [47]. In mixed genetic backgrounds, many virgin p75 CUX1 mice succumbed to myeloproliferative-disease (MPD)-like myeloid leukemias [41]. In the FVB genetic background, multiparous transgenic mice carrying a MMTV-p75 or MMTV-p110 CUX1 transgene developed malignancies primarily in the mammary gland [42]. In addition to mammary tumors, a small number of tumors were also detected in other tissues. In particular, a number of sarcomas with features resembling that of histiocytic sarcomas were observed in the uterus and liver of several mice. An example of this type of tumor is presented (Fig. 4). The uterus was grossly enlarged (Fig. 4B), and a large number of histiocytic/dendritic cells were observed in sections taken from the uterus and the liver (Fig. 4D and 4F). Overall, results from CUX1 transgenic mice indicate that overexpression of p110 or p75 CUX1 can increases the incidence of cancer in various cell types.

**Figure 5:**
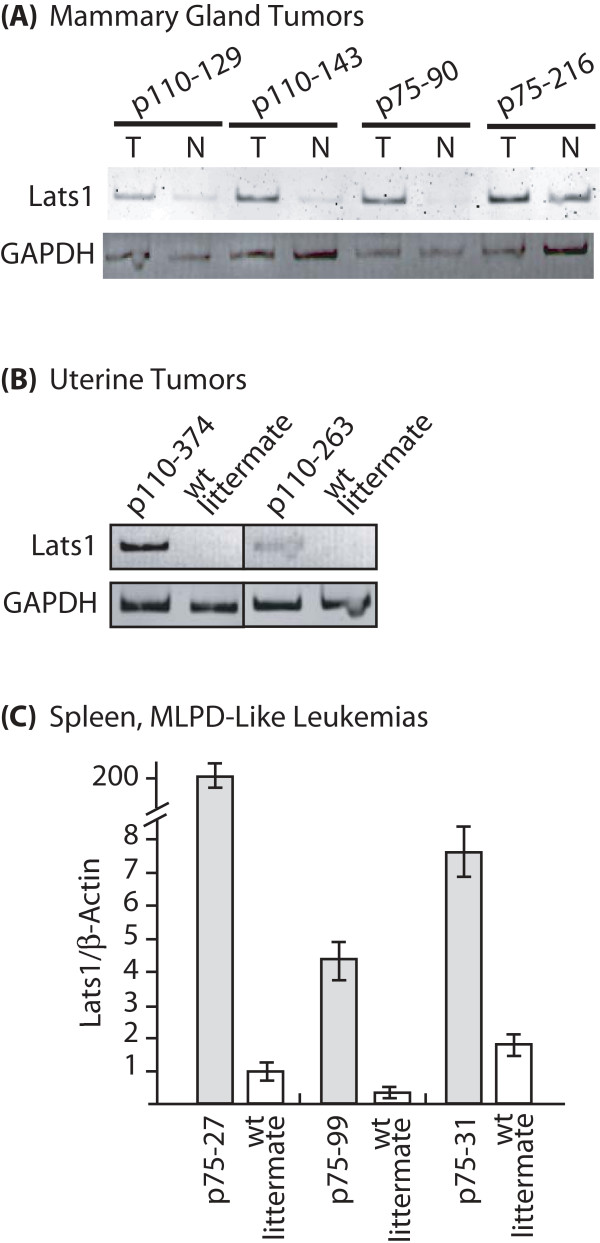
***Lats1 *is overexpressed in tumors from p75 and p110 CUX1 transgenic mice**. RNA was prepared from tumors and either adjacent normal tissue (mammary gland) or equivalent normal tissue from a non-transgenic littermate. *Lats1 *mRNA expression was analyzed by RT-PCR using GAPDH or β-actin as a control, as indicated. (A) Mammary gland tumors and adjacent normal mammary gland. (B) Uterine tumors and normal uterus from a non-transgenic littermate. (C) Enlarged and normal spleens from p75 CUX1 transgenic mice and non-transgenic littermates, respectively.

We analyzed *Lats1 *mRNA expression in 4 mammary gland tumors, 2 uterine tumors and 3 spleens from mice suffering from MLPD-like myeloid leukemias. In the case of mammary tumors, RNA from an adjacent normal mammary gland served as a control. In the case of uterine tumors and diseased spleens, we used RNA from the corresponding normal tissue of a non transgenic littermate. In all cases, *Lats1 *mRNA expression was increased in tumors from CUX1 transgenic mice (Fig. 5A, B and 5C).

### Overexpression of *Lats1 *in NMuMG mammary epithelial cells does not hinder cell cycle progression

**Figure 6:**
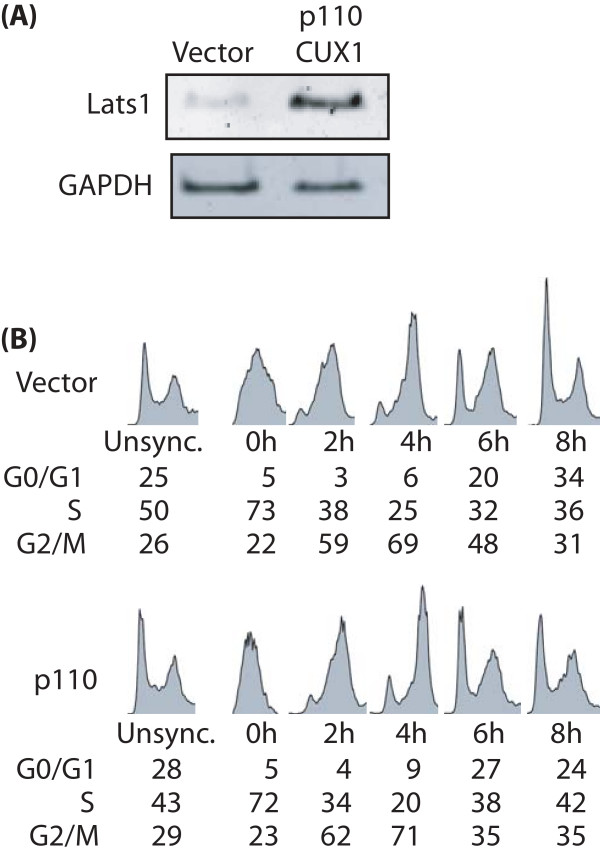
**Overexpression of *Lats1 *in NMuMG epithelial cells does not hinder cell cycle progression**. (A) RNA was prepared from NMuMG mouse epithelial cells expressing p110 CUX1 or carrying an empty vector. *Lats1 *mRNA expression was analyzed by RT-PCR using GAPDH as a control. (B) Cells were synchronized at the G1/S transition using the double thymidine block procedure, and then released from the block and allowed to progress in the cell cycle. Nuclei were stained with propidium iodide and DNA content was determined by fluorescence-activated cell sorting (FACS) analysis. Cell cycle profiles were analyzed using the Watson model and the FlowJo™ software. The numbers below the FACS profiles indicate the percentage of cells with 2N DNA content (G0/G1 cells), between 2N and 4N DNA content (S phase cells) and 4N DNA content (G2/M cells).

Loss of *Lats1 *and its ortholog warts has been associated with increased susceptibility to various types of cancers [2,3,6]. This protective function of *Lats1 *against cancer has been linked to its role in cell cycle progression during mitosis. However, it is not clear from previous studies whether overexpression of *Lats1 *would necessarily cause a delay in cell cycle progression [21,24]. We therefore investigated cell cycle progression using non-transformed NMuMG mouse mammary epithelial cells carrying a p110 CUX1 retroviral vector or the empty vector. As shown in Fig. 6A, *Lats1 *expression was significantly higher in cells carrying the p110 CUX1 retrovirus. Cells were synchronized at the G1/S transition using the double thymidine block procedure and then allowed to proceed through the cell cycle. Samples taken at different times were stained with propidium iodide and the cell cycle profile of cells was analyzed by FACS analysis. This experiment was performed independently three times and representative results are presented (Fig. 6B). No delay in cell cycle progression was observed in cells over-expressing p110 CUX1 and *Lats1*. In fact, cells of the p110 CUX1 population reached the next G1 phase a little bit faster than the control vector-cells (Fig. 6B, see the 6-hour time point). We conclude that higher expression of *Lats1 *does not slow down progression through the S, G2 and mitosis phases of the cell cycle.

## Discussion

We have presented evidence demonstrating that the *Lats1 *gene is a transcriptional target of the CUX1 homeodomain protein. The *Lats1 *gene promoter region was identified in 8 of 8 genome-wide location arrays using CUX1-associated chromatin from cell lines of various tissue types (Table 1). Scanning ChIP analysis confirmed that CUX1 binds to genomic sequences located immediately upstream of the *Lats1 *transcription start site and, to a lesser extent, to sequences within the first intron (Fig. 1). The 5' flanking sequences were found to include two CUX1 consensus binding sites, and the intron 1 sequences included one such binding site (Fig. 1). A luciferase reporter plasmid containing the immediate promoter region of *Lats1 *was activated by p110 and p75 CUX1 (Fig. 2). Comparison of *Lats1 *steady state mRNA levels in cux1^-/- ^and cux1^+/+ ^MEFs indicated that CUX1 is required for *Lats1 *expression (Fig. 3A). Indeed, *Lats1 *expression was stimulated up to 15-fold upon the reintroduction of p110 CUX1 in cux1^-/- ^MEFs (Fig. 3B). Interestingly, in cell lines that are not defective for CUX1, *Lats1 *expression was also stimulated following the infection of cells with a retrovirus expressing p110 or p75 CUX1 (Fig. 3B, C and 3D and 6A). As CUX1 was shown to participate in the transcriptional activation of several genes that play a role in DNA replication and mitosis, the regulation of *Lats1 *by CUX1 is consistent with the function of CUX1 in cell cycle progression [35]. CUX1 DNA binding and transcriptional activities were previously shown to be activated at the end of the G1 phase [30,31,43]. Once activated, CUX1 cooperates with other factors to activate the transcription of genes that are required for the replication of DNA and later the completion of cell division [34-36,46]. Constitutive expression of p110 CUX1 was indeed shown to accelerate entry into S phase without apparent deleterious consequences [36]. Cells continued to progress unabated through the cell cycle and proliferated faster and reached a higher density at saturation than control cells [36].

Transgenic mice expressing p75 or p110 CUX1 developed tumors in various tissues including the hematopoietic system, the mammary gland and the uterus. In mammary tumors, in MLPD-like leukemias and in uterine tumors, *Lats1 *mRNA expression was consistently elevated in tumors as compared to control normal tissues (Fig. 5). Although this finding is consistent with the fact that CUX1 activates *Lats1 *transcription, there is an apparent discrepancy between the elevated expression of *Lats1 *in tumors from transgenic mice and the purported role of LATS1 as a tumor suppressor. Obviously, tumors can grow in spite of elevated LATS1 expression. Moreover, when cell cycle progression was monitored in tissue culture, we did not observe any delay in cells that over-expressed both p110 CUX1 and *Lats1 *(Fig. 6). In this respect, our results are in agreement with a previous study showing that a moderate increase (from 2 to 5-fold) in *Lats1 *expression did not hinder cell cycle progression [24]. In contrast, the introduction of a *Lats1 *gene into cells by Adenovirus-mediated gene transfer caused a block at the G2/M transition [21]. We favor the interpretation proposed by Bothos et al. suggesting that the extremely high concentration of *Lats1 *that was achieved with an Adenovirus vector may have caused the premature inhibition of cyclin B/Cdk1 at the end of the G2 phase, instead of during telophase as in normal cells [21,24].

There is solid genetic evidence in Drosophila and mice to establish the role of LATS1 as a tumor suppressor. Loss-of-function of warts in Drosophila and of *Lats1 *in the mouse has been shown to increase the susceptibility to cancers in various tissues [3,4,6]. Yet, the role of LATS1 as a tumor suppressor does not necessarily mean that elevated expression of LATS1 would hinder cell proliferation and prevent tumor growth, as demonstrated here and in another study [24]. Moreover, genetic analysis in human tumors did not provide a compelling case for the inactivation of *LATS1 *in human cancers. While hypermethylation of the *LATS1 *promoter has been reported [9-11], these cases are not common and so far no inactivating mutation has been documented. Interestingly, a search of the Oncomine database revealed only two studies where a difference in *LATS1 *expression was observed between normal and cancer samples [48]. In both cases, cervical cancers and basal-like breast cancers, *LATS1 *expression was higher in the tumor than in the normal tissue [49,50].

## Conclusion

In summary, while loss-of-function of *Lats1 *can predispose to cancer, there is compelling evidence both from transgenic mouse models and from human cancers that *Lats1 *can be overexpressed in tumors. *Lats1 *is not unique in this respect. Indeed, many genes that play a role in the mitotic checkpoint were found to be either over-expressed or inactivated in various cancers [51,52]. While the mechanistic details remain to be deciphered, the overall picture that has emerged suggests that the mitotic regulatory network can be perturbed through both the elevated or decreased expression of some of its components (reviewed in [52]).

## Methods

### Cell Culture

NIH3T3, Hs578T and NMuMG cells were cultured in Dulbecco's Modified Minimum Essential Medium (DMEM) supplemented with penicillin-streptomycin, glutamine, and 10% fetal bovine serum (FBS) (Gibco); 10 μg/ml insulin was added to the medium for NMuMG cells.

### Cell synchronization and Fluorescence -activated cell sorting (FACS) analysis

NMuMG cells (100,000 cell/plate) were cultured in DMEM medium supplemented with 10% FBS and 10 μg/ml insulin. Synchronization in G1/S was performed using the double thymidine procedure [53]. Cells were cultured overnight in DMEM plus 10% FBS supplemented with 2 mM thymidine, washed the next day, cultured for 10 hours in DMEM plus 10% FBS and finally further incubated overnight in the presence of 2 mM thymidine (Thymidine 0 h). To allow cells to progress in the cell cycle, the medium was replaced with DMEM plus 10% FBS and cells were harvested at different times thereafter. FACS analysis was performed as previously described [43].

### Chromatin Immunoprecipitation

We used 4 × 10^8 ^Hs578T cells for the chromatin immunoprecipitation with anti CUX1 antibodies 861 and 1300, using our previously described protocol [34,35]. Chromatin affinity purification (ChAP) was performed for the genome-wide location analysis. A complete protocol for ChAP as well as an excel file detailing the results in 8 cell lines are provided in the supplemental data of Harada et al., 2008 [35]. For scanning ChiP, the following primers were used: primer that amplify region A, 5'-TTGGGTTTGATGTATAAGGTACGG-3' and 5'-GTGCTGGGATTACAGGCGTGAGAC-3'; region B, 5'-TCTTATCTGTCAACCGCATCCGTAGA-3' and 5'-GGACCGTACCTTATACATCAAACCCAACGA-3'; region C, 5'-AAAGTGTTAATTTGGGTTGAA-3' and 5'-CATGAAGATACAGCGTTGCTT-3'; region D, 5'-AAAGTGTTAATTTGGGTTGAA-3' and 5'-CATGAAGATACAGCGTTGCTT-3'; region E, 5'-CAGGCTGGAATTGGG TATCTTATT-3'and 5'-ATG GGAAACTGGAGGTGGGCTAAC-3'

### Luciferase construct and luciferase assay

*LATS1 *5' flanking sequences were amplified from Hs578T genomic DNA using the following primers 5'-ATCATTCCACCTCTGCTTTCCCTGC-3' and 5'-TGGGGCAGAGCGGGGAGACG-3' which amplifies a 1775 base pair fragment that include 1464 base pair upstream of the transcription start site, start site and 311 base pairs of exon 1. The PCR product was cloned into XhoI and HindIII site of pGL3 (Promega). NIH3T3 cells and Hs578T cells were transiently transfected with CUX1 p200, p110 (831–1505) or p75 (1062–1505) and pGL3-*Lats1*. We utilized 2 different vectors for the CUX1 constructs pTriEx1.1 (Novagen) and pXJ42 [54], both revealing similar results. Luciferase assay was performed as previously described [30].

### RNA extraction, reverse transcriptase PCR (RT-PCR) and real-time PCR

RNA was extracted using TRIzol reagent (Invitrogen) followed by cDNA preparation using the superscript II RNase H-reverse transcriptase kit (Invitrogen) as provided in the manufacturer manual. Real-time PCR was performed on a LightCycler instrument (Roche) using the FastStart DNA Master SYBR Green kit (Roche). The following oligonucleotides were used for the amplification of mouse *Lats1 *cDNA: 5'-TAGAATGGGCATCTTTCCTGA-3' and 5'-TGCTATCTTGCCGTGGGT-3' (Fig. 3A and 3D), and 5'-AATAGGAGCGTTTGGTGAAGTC-3' and 5'-TGTGTCCATCTGAAGCCAGTG-3' (Fig. 5). Human *LATS1 *cDNA: 5'-ACCGCTTCAAATGTGACTGTGATGCCACCT-3' and 5'-CTTCCTTGGGCAAGCTTGGCTGATCCTCT-3'.

## Competing interests

The authors declare that they have no competing interests.

## Authors' contributions

RS carried out scanning ChIP, luciferase assays, RT-PCR assays and drafted the manuscript. RH carried out the ChIP-chip assays and the cell cycle progression studies. CC analyzed tumors in CUX1 transgenic mice. RB carried RT-PCR assays. CV performed statistical analysis of ChIP-chip data. AN conceived of the study, and participated in its design and coordination and helped to draft the manuscript. All authors read and approved the final manuscript.

## References

[B1] Zeng Q, Hong W (2008). The emerging role of the hippo pathway in cell contact inhibition, organ size control, and cancer development in mammals. Cancer Cell.

[B2] Turenchalk GS, St John MA, Tao W, Xu T (1999). The role of lats in cell cycle regulation and tumorigenesis. Biochim Biophys Acta.

[B3] Xu T, Wang W, Zhang S, Stewart RA, Yu W (1995). Identifying tumor suppressors in genetic mosaics: the Drosophila lats gene encodes a putative protein kinase. Development (Suppl).

[B4] Justice RW, Zilian O, Woods DF, Noll M, Bryant PJ (1995). The Drosophila tumor suppressor gene warts encodes a homolog of human myotonic dystrophy kinase and is required for the control of cell shape and proliferation. Genes Dev.

[B5] Tao W, Zhang S, Turenchalk GS, Stewart RA, St John MA, Chen W, Xu T (1999). Human homologue of the Drosophila melanogaster lats tumour suppressor modulates CDC2 activity. Nat Genet.

[B6] St John MA, Tao W, Fei X, Fukumoto R, Carcangiu ML, Brownstein DG, Parlow AF, McGrath J, Xu T (1999). Mice deficient of Lats1 develop soft-tissue sarcomas, ovarian tumours and pituitary dysfunction. Nat Genet.

[B7] Morinaga N, Shitara Y, Yanagita Y, Koida T, Kimura M, Asao T, Kimijima I, Takenoshita S, Hirota T, Saya H, Kuwano H (2000). Molecular analysis of the h-warts/LATS1 gene in human breast cancer. Int J Oncol.

[B8] Rutherford S, Yu Y, Rumpel CA, Frierson HF, Moskaluk CA (2006). Chromosome 6 deletion and candidate tumor suppressor genes in adenoid cystic carcinoma. Cancer Lett.

[B9] Jiang Z, Li X, Hu J, Zhou W, Jiang Y, Li G, Lu D (2006). Promoter hypermethylation-mediated down-regulation of LATS1 and LATS2 in human astrocytoma. Neuroscience Research.

[B10] Takahashi Y, Miyoshi Y, Takahata C, Irahara N, Taguchi T, Tamaki Y, Noguchi S (2005). Down-regulation of LATS1 and LATS2 mRNA expression by promoter hypermethylation and its association with biologically aggressive phenotype in human breast cancers. Clin Cancer Res.

[B11] Hisaoka M, Tanaka A, Hashimoto H (2002). Molecular alterations of h-warts/LATS1 tumor suppressor in human soft tissue sarcoma. Lab Invest.

[B12] Wu S, Huang J, Dong J, Pan D (2003). hippo encodes a Ste-20 family protein kinase that restricts cell proliferation and promotes apoptosis in conjunction with salvador and warts. Cell.

[B13] Dong J, Feldmann G, Huang J, Wu S, Zhang N, Comerford SA, Gayyed MF, Anders RA, Maitra A, Pan D (2007). Elucidation of a universal size-control mechanism in Drosophila and mammals. Cell.

[B14] Huang J, Wu S, Barrera J, Matthews K, Pan D (2005). The Hippo signaling pathway coordinately regulates cell proliferation and apoptosis by inactivating Yorkie, the Drosophila Homolog of YAP. Cell.

[B15] Chan EH, Nousiainen M, Chalamalasetty RB, Schafer A, Nigg EA, Sillje HH (2005). The Ste20-like kinase Mst2 activates the human large tumor suppressor kinase Lats1. Oncogene.

[B16] Hao Y, Chun A, Cheung K, Rashidi B, Yang X (2008). Tumor suppressor LATS1 is a negative regulator of oncogene YAP. JBC.

[B17] Nishiyama Y, Hirota T, Morisaki T, Hara T, Marumoto T, Iida S, Makino K, Yamamoto H, Hiraoka T, Kitamura N, Saya H (1999). A human homolog of Drosophila warts tumor suppressor, h-warts, localized to mitotic apparatus and specifically phosphorylated during mitosis. FEBS Lett.

[B18] Morisaki T, Hirota T, Iida S, Marumoto T, Hara T, Nishiyama Y, Kawasuzi M, Hiraoka T, Mimori T, Araki N (2002). WARTS tumor suppressor is phosphorylated by Cdc2/cyclin B at spindle poles during mitosis. FEBS Lett.

[B19] Hirota T, Morisaki T, Nishiyama Y, Marumoto T, Tada K, Hara T, Masuko N, Inagaki M, Hatakeyama K, Saya H (2000). Zyxin, a regulator of actin filament assembly, targets the mitotic apparatus by interacting with h-warts/LATS1 tumor suppressor. J Cell Biol.

[B20] Yang X, Yu K, Hao Y, Li DM, Stewart R, Insogna KL, Xu T (2004). LATS1 tumour suppressor affects cytokinesis by inhibiting LIMK1. Nat Cell Biol.

[B21] Yang X, Li DM, Chen W, Xu T (2001). Human homologue of Drosophila lats, LATS1, negatively regulate growth by inducing G(2)/M arrest or apoptosis. Oncogene.

[B22] Xia H, Qi H, Li Y, Pei J, Barton J, Blackstad M, Xu T, Tao W (2002). LATS1 tumor suppressor regulates G2/M transition and apoptosis. Oncogene.

[B23] Tamaskovic R, Bichsel SJ, Hemmings BA (2003). NDR family of AGC kinases – essential regulators of the cell cycle and morphogenesis. FEBS Lett.

[B24] Bothos J, Tuttle RL, Ottey M, Luca FC, Halazonetis TD (2005). Human LATS1 is a mitotic exit network kinase. Cancer Res.

[B25] Nepveu A (2001). Role of the multifunctional CDP/Cut/Cux homeodomain transcription factor in regulating differentiation, cell growth and development. Gene.

[B26] Sansregret L, Nepveu A (2008). The multiple roles of CUX1: Insights from mouse models and cell-based assays. Gene.

[B27] Moon NS, Berube G, Nepveu A (2000). CCAAT displacement activity involves Cut repeats 1 and 2, not the Cut homeodomain. JBC.

[B28] Barberis A, Superti-Furga G, Busslinger M (1987). Mutually exclusive interaction of the CCAAT-binding factor and of a displacement protein with overlapping sequences of a histone gene promoter. Cell.

[B29] Neufeld EJ, Skalnik DG, Lievens PM, Orkin SH (1992). Human CCAAT displacement protein is homologous to the Drosophila homeoprotein, cut. Nature Genetics.

[B30] Moon NS, Premdas P, Truscott M, Leduy L, Berube G, Nepveu A (2001). S Phase-Specific Proteolytic Cleavage Is Required to Activate Stable DNA Binding by the CDP/Cut Homeodomain Protein. MCB.

[B31] Goulet B, Baruch A, Moon NS, Poirier M, Sansregret LL, Erickson A, Bogyo M, Nepveu A (2004). A Cathepsin L Isoform that Is Devoid of a Signal Peptide Localizes to the Nucleus in S Phase and Processes the CDP/Cux Transcription Factor. Mol Cell.

[B32] Goulet B, Sansregret L, Leduy L, Bogyo M, Weber E, Chauhan SS, Nepveu A (2007). Increased expression and activity of nuclear cathepsin L in cancer cells suggests a novel mechanism of cell transformation. Molecular Cancer Research.

[B33] Goulet B, Watson P, Poirier M, Leduy L, Berube G, Meterissian S, Jolicoeur P, Nepveu A (2002). Characterization of a tissue-specific CDP/Cux isoform, p75, activated in breast tumor cells. Cancer Research.

[B34] Truscott M, Raynal L, Premdas P, Goulet B, Leduy L, Berube G, Nepveu A (2003). CDP/Cux stimulates transcription from the DNA polymerase alpha gene promoter. Molecular and Cellular Biology.

[B35] Harada R, Vadnais C, Sansregret L, Leduy L, Berube G, Robert F, Nepveu A (2008). Genome-wide location analysis and expression studies reveal a role for p110 CUX1 in the activation of DNA replication genes. Nucleic Acids Res.

[B36] Sansregret L, Goulet B, Harada R, Wilson B, Leduy L, Bertoglio J, Nepveu A (2006). The p110 isoform of the CDP/Cux transcription factor accelerates entry into S phase. Mol Cell Biol.

[B37] De Vos J, Thykjaer T, Tarte K, Ensslen M, Raynaud P, Requirand G, Pellet F, Pantesco V, Reme T, Jourdan M (2002). Comparison of gene expression profiling between malignant and normal plasma cells with oligonucleotide arrays. Oncogene.

[B38] Tsutsumi S, Taketani T, Nishimura K, Ge X, Taki T, Sugita K, Ishii E, Hanada R, Ohki M, Aburatani H, Hayashi Y (2003). Two distinct gene expression signatures in pediatric acute lymphoblastic leukemia with MLL rearrangements. Cancer Res.

[B39] Michl P, Ramjaun AR, Pardo OE, Warne PH, Wagner M, Poulsom R, D'Arrigo C, Ryder K, Menke A, Gress T, Downward J (2005). CUTL1 is a target of TGF(beta) signaling that enhances cancer cell motility and invasiveness. Cancer Cell.

[B40] Moon NS, Rong Zeng W, Premdas P, Santaguida M, Berube G, Nepveu A (2002). Expression of N-terminally truncated isoforms of CDP/CUX is increased in human uterine leiomyomas. International Journal of Cancer.

[B41] Cadieux C, Fournier S, Peterson AC, Bédard C, Bedell BJ, Nepveu A (2006). Transgenic Mice Expressing the p75 CCAAT-Displacement Protein/Cut Homeobox Isoform Develop a Myeloproliferative Disease-Like Myeloid Leukemia. Cancer Res.

[B42] Cadieux C, Kedinger V, Yao L, Vadnais C, Drossos M, Paquet M, Nepveu A MMTV-p75 and p110 CUX1 Transgenic Mice Develop Mammary Tumors of Various Histological Types. Cancer Research.

[B43] Coqueret O, Berube G, Nepveu A (1998). The mammalian Cut homeodomain protein functions as a cell-cycle dependent transcriptional repressor which downmodulates p21WAF1/CIP1/SDI1 in S phase. EMBO Journal.

[B44] Laganiere J, Deblois G, Lefebvre C, Bataille AR, Robert F, Giguere V (2005). From the Cover: Location analysis of estrogen receptor alpha target promoters reveals that FOXA1 defines a domain of the estrogen response. Proc Natl Acad Sci USA.

[B45] Harada R, Berube G, Tamplin OJ, Denis-Larose C, Nepveu A (1995). DNA-binding specificity of the cut repeats from the human cut-like protein. MCB.

[B46] Truscott M, Harada R, Vadnais C, Nepveu A (2008). The p110 isoform of CUX1 Cooperates with E2F Transcription Factors in the Transcriptional Activation of Cell Cycle-Regulated Gene Promoters. Molecular and Cellular Biology.

[B47] Bronson SK, Plaehn EG, Kluckman KD, Hagaman JR, Maeda N, Smithies O (1996). Single-copy transgenic mice with chosen-site integration [see comments]. Proc Natl Acad Sci USA.

[B48] Rhodes DR, Yu J, Shanker K, Deshpande N, Varambally R, Ghosh D, Barrette T, Pandey A, Chinnaiyan AM (2004). ONCOMINE: a cancer microarray database and integrated data-mining platform. Neoplasia.

[B49] Richardson AL, Wang ZC, De Nicolo A, Lu X, Brown M, Miron A, Liao X, Iglehart JD, Livingston DM, Ganesan S (2006). X chromosomal abnormalities in basal-like human breast cancer. Cancer Cell.

[B50] Pyeon D, Newton MA, Lambert PF, den Boon JA, Sengupta S, Marsit CJ, Woodworth CD, Connor JP, Haugen TH, Smith EM (2007). Fundamental differences in cell cycle deregulation in human papillomavirus-positive and human papillomavirus-negative head/neck and cervical cancers. Cancer Res.

[B51] Weaver BA, Cleveland DW (2006). Does aneuploidy cause cancer?. Curr Opin Cell Biol.

[B52] Weaver BA, Silk AD, Montagna C, Verdier-Pinard P, Cleveland DW (2007). Aneuploidy acts both oncogenically and as a tumor suppressor. Cancer Cell.

[B53] Stein GS, Borun TW (1972). The synthesis of acidic chromosomal proteins during the cell cycle of HeLa S-3 cells. I. The accelerated accumulation of acidic residual nuclear protein before the initiation of DNA replication. JCB.

[B54] Mengus G, May M, Carre L, Chambon P, Davidson I (1997). Human TAF(II)135 potentiates transcriptional activation by the AF-2s of the retinoic acid, vitamin D3, and thyroid hormone receptors in mammalian cells. Genes Dev.

